# Left Main Coronary Artery Disease: A Contemporary Review of Diagnosis and Management

**DOI:** 10.31083/j.rcm2502066

**Published:** 2024-02-18

**Authors:** Muzamil Khawaja, Michael Britt, Muhammad Asad Khan, Uzair Munaf, Hassaan Arshad, Rehma Siddiqui, Hafeez Ul Hassan Virk, Mahboob Alam, Chayakrit Krittanawong

**Affiliations:** ^1^Department of Cardiology, Emory University, Atlanta, GA 30322, USA; ^2^Department of Internal Medicine, Emory University, Atlanta, GA 30322, USA; ^3^Department of Family Medicine, West Chicago Immediate Care, West Chicago, IL 60185-2847, USA; ^4^Department of Internal Medicine, Dow Medical College, 75300 Karachi, Pakistan; ^5^Department of Internal Medicine, Capital Health Regional Medical Centre, Trenton, NJ 08638-4143, USA; ^6^Department of Internal Medicine, The University of Mississippi Medical Center, Jackson, MS 39216, USA; ^7^Harrington Heart & Vascular Institute, Case Western Reserve University, University Hospitals Cleveland Medical Center, Cleveland, OH 44106-2333, USA; ^8^Department of Cardiology, The Texas Heart Institute, Baylor College of Medicine, Houston, TX 77030, USA; ^9^Cardiology Division, NYU Langone Health and NYU School of Medicine, New York, NY 10016, USA

**Keywords:** left main coronary artery disease, percutaneous coronary intervention, coronary artery bypass grafting, revascularization

## Abstract

Significant left main coronary artery disease is a very high-risk subgroup of coronary artery disease that is a crucial indicator of heightened morbidity and mortality rates. Despite its clinical significance, uncertainties persist regarding the optimal management strategy for patients, particularly given its phenotypic variations. Existing evidence-based guidelines offer insights into revascularization options, yet questions remain regarding long-term prognoses and clinical outcomes when comparing percutaneous coronary intervention to coronary artery bypass grafting. This comprehensive review aims to provide an in-depth analysis of contemporary strategies for the diagnosis, assessment, and treatment of left main coronary artery disease. By synthesizing current literature and addressing the evolving landscape of revascularization modalities, this review seeks to contribute valuable insights for clinicians and researchers grappling with the complexities of managing left main coronary artery disease.

## 1. Introduction of Left Main Coronary Disease

The left main coronary artery supplies blood flow to most of the left ventricular myocardium. As such, significant left main coronary artery disease (LMCAD) is the highest-risk lesion subgroup among the different types of obstructive coronary artery disease (CAD) and is associated with poorer outcomes [1]. It is seen in roughly 5%–7% of all patients that undergo coronary angiography for an ischemic evaluation [2]. Multiple studies have found LMCAD to be an independent indicator of increased morbidity and mortality rates among patients with CAD [3, 4]. As such, this disease has significant implications for population-wide cardiovascular morbidity and mortality. While the clinical significance of LMCAD is unquestionable, its best management strategy remains uncertain. Management becomes especially complex when the phenotypic variation of LMCAD is taken into consideration. Coronary artery bypass grafting (CABG) had previously been the mainstay of revascularization for the management of significant, symptomatic LMCAD. However, with progressive advancements in its design and feasibility, percutaneous coronary intervention (PCI) has emerged as a useful revascularization option. Current evidence-based guidelines offer insight into the options for revascularization, but a lot is unknown regarding overall long-term prognoses and clinical outcomes when comparing PCI to CABG for many of these patients. As such, this review aims to provide an in-depth analysis of contemporary strategies for the diagnosis, assessment, and treatment of LMCAD.

## 2. Clinical Presentation and Symptoms

Like lesions affecting other coronary arteries, LMCAD can lead to ischemia involving a large portion of the myocardium. As a result, clinical manifestations are consistent with angina and other ischemia-associated symptoms. The acuity and degree of symptoms is likely related to lesion severity and overall coronary anatomy, with clinical manifestations ranging from asymptomatic disease to sudden cardiac death. Cardiac chest pain is typically described as a retrosternal pressure or tightness, classically provoked by exertion, and resolving with rest or nitroglycerin [5]. Common associated symptoms include pain radiation to the neck, arm or jaw, shortness of breath, and nausea. While these symptoms are common in the setting of CAD, there is limited data characterizing clinical presentations specific to LMCAD. In a retrospective study of 127 patients in the 1980s with significant LMCAD, 85% of patients presented with typical anginal symptoms and 65% presented with unstable angina [6]. More recent data demonstrates significant LMCAD may be found in 4 to 6% of patients that undergo coronary angiography and is present in about 24% of patients presenting with acute coronary syndromes [7, 8]. Only 44% of patients in the study presented with dyspnea. It’s also worth noting that clinical presentation is often atypical in female patients, but chest pain is still the most common symptom of disease [5].

Risk factors for LMCAD are the same as traditional risk factors for CAD, including hypertension, hypercholesterolemia, diabetes, tobacco use and obesity [9]. In fact, Senior *et al*. [10] performed a post-hoc analysis of the Initial Invasive or Conservative Strategy for Stable Coronary Disease (ISCHEMIA) trial to assess for risk factors specific to patients with evidence of LMCAD on coronary computed tomography angiography (CCTA). Older age was significantly associated with a higher probability of LMCAD, with an odds ratio of 1.42 (95% CI 1.21–1.66) at age 65 and 1.56 (95% CI 1.21–2.01) at age 75 when compared to patients 55 years old at study enrollment [10] Conversely, female sex and prior myocardial infarction (MI) were both associated with lower odds of LMCAD, with odds ratios of 0.32 and 0.61, respectively [10].

## 3. Diagnosis of Left Main Disease

The diagnosis of hemodynamically significant LMCAD is essential to determine an overall management strategy. Several diagnostic techniques have been validated for the early detection and diagnosis of LMCAD, which are highlighted in this section and summarized in Table 1.

**Table 1.** **Advantages and disadvantages of diagnostic tools for LMCAD**.

Diagnostic method	Advantages	Disadvantages
ECG	Quick and noninvasive	Limited sensitivity and specificity
	Useful to roughly localize	Dependence on ischemic changes
	Ischemia	Unable to visualize coronary anatomy
	Assess for dynamic changes	Susceptible to influence by other factors (meds, electrolytes, other conditions)
	Widely available	Variability among individuals
TTE/TEE	Non-invasive	Limited visualization of coronaries
	Widely accessible	Limited diagnostic accuracy
	Dynamic imaging	Invasive nature (TEE)
		Risk of procedural complications (TEE)
		Requires specialized training to perform and interpret
CCTA	Non-invasive imaging	Radiation exposure
	High spatial resolution	Contrast agent use
	Assessment of coronary anatomy	Calcium blooming artifact
	Evaluation of extracardiac structures	Limited functional information
		Limited in certain patient populations (high heart rate, obesity, etc.)
Angiography	High spatial resolution	Invasive
	Real-time imaging	Contrast agent use
	Functional assessment (FFR)	Radiation
	Means for diagnosis and therapy	Limited assessment of plaque characteristics
	Direct visualization of anatomy	

ECG, electrocardiogram; TTE, transthoracic echocardiography; TEE, transesophageal echocardiography; CCTA, coronary computed tomography angiography; LMCAD, left main coronary artery disease; FFR, fractional flow reserve.

### 3.1 Electrocardiogram 

When assessed with an electrocardiogram (ECG), acute coronary syndrome involving LMCAD classically presents with ST-segment elevation (STE) in lead augmented vector right (aVR), along with diffuse ST depressions, most prominently in leads I, II and V4-V6 [11, 12]. There are two mechanisms to explain STE in lead aVR: (a) diffuse subendocardial ischemia, with ST depression in the lateral leads producing reciprocal changes in aVR or (b) infarction of the basal septum. Notably, the lack of STE in aVR is highly sensitive to exclude a significant left main lesion for an acute coronary syndrome. Several studies have evaluated the electrocardiographic finding of STE in lead aVR as a diagnostic predictor of LMCAD. Yamaji *et al*. [11] retrospectively evaluated ECGs of 86 patients with either left main, left anterior descending (LAD) or right coronary artery (RCA) disease and found that STE in lead aVR greater than V1 was able to distinguish left main and LAD culprit lesions 81% of the time (81% sensitivity, 80% specificity). They were able differentiate LMCAD from RCA disease with a finding of STE in aVR with 90% accuracy (88% sensitivity, 92% specificity), noting that the presence of STE in inferior leads was also beneficial to make this diagnosis [11]. A meta-analysis by Lee *et al*. [13] established that STE greater than or equal to 0.05 mV in lead aVR had an odds ratio of 6.64 (95% CI: 4.80–9.17) for LMCAD. In addition, a retrospective, single center study of 572 patients in Japan admitted with cardiac chest pain demonstrated that STE of 1.0 mm or greater in lead aVR had an odds ratio of 29.1 (95% CI 9.54–49.8) for left main and/or three vessel disease, highlighting an increase in specificity of this finding with the degree of STE [14]. An additional study by Kosuge *et al*. [15] on STE of ≥0.5 mm in aVR found a significantly high odds ratio of 12.8 (95% CI 4.80–33.9) for the primary endpoint of MI, death, or urgent revascularization at 90 days. A table summarizing these studies on ECG patterns in acute coronary syndrome secondary to LMCAD is presented below (Table 2, Ref. [11, 13, 14, 15]). However, it is worth noting that LMCAD presenting with chronic stable angina or asymptomatic disease may not have overt electrocardiogram changes, thereby complicating diagnosis. While some patients with chronic LMCAD may remain asymptomatic, subtle ECG changes can be observed. These changes may include variations in the ST segment or T-wave abnormalities, reflecting the myocardial response to the chronic ischemic condition. However, it is important to note that ECG findings can be nonspecific, and the absence of typical symptoms further complicates the identification of LMCAD solely based on electrocardiographic changes.

**Table 2.** **Studies on ECG changes associated with LMCAD MI**.

Study (Authors)	Year	ECG findings	Results
Yamaji *et al*. [11]	2001	The finding of lead aVR ST-segment elevation greater than or equal to lead V1 ST-segment elevation distinguished the acute occlusion in LMCAD group from the LAD group	81% sensitivity
			80% specificity
Kuo Lee *et al*. [13]	2019	ST-segment elevation ≥0.05 mV was associated with a higher incidence rate of LMCAD	STE ≥0.05 mV association with LMCAD: OR = 6.64, 95% CI: 4.80–9.17
		The degree of STE in lead aVR was significantly associated with LMCAD	
Kosuge *et al*. [14]	2011	ST-segment elevation ≥1.0 mm in lead aVR on admission electrocardiogram is highly suggestive of severe LMCAD (>75%) in patients with non ST-elevation MI	80% sensitivity, 93% specificity, 56% positive predictive value, and 98% negative predictive value
Kosuge *et al*. [15]	2006	ST-segment elevation of ≥0.5 mm in lead aVR combined with troponin T had the highest rates of left main or 3-vessel coronary disease (62%) and 90-day adverse outcomes (47%)	STE ≥0.5 mm and elevated troponin T
			Rate of LMCAD or 3 vessel CAD: 62%
			Rate of 90 day adverse outcomes: 47%

aVR, lead augmented vector right; LMCAD, left main coronary artery disease; ECG, electrocardiogram; LAD, left anterior descending; MI, myocardial infarction; STE, ST-segment elevation; OR, odds ratio; CAD, coronary artery disease.

There are important limitations to many of the aforementioned studies on the diagnosis of LMCAD with ECG. Firstly, there is significant heterogeneity of the patient populations studied, which ultimately impacts the generalizability of study results. Secondly, LMCAD is relatively uncommon when compared to other types of CAD. Such low prevalence impacts the statistical power of studies. Some studies also rely on resting ECG alone when assessing for LMCAD. However, the sensitivity of resting ECG may be limited, especially in cases of inducible ischemia. Moreover, many of these studies are single-center studies which also limits generalizability. Lastly, there is the possibility of interpretation variability amongst providers. Differences in expertise and familiarity with ECG changes can impact the sensitivity of ECG for LMCAD.

### 3.2 Echocardiogram

The diagnosis of LMCAD through echocardiography represents a non-invasive and valuable tool in the assessment of coronary artery pathology. Echocardiography, specifically transthoracic echocardiography (TTE) and transesophageal echocardiography (TEE), enables clinicians to visualize and evaluate the left main coronary artery and its branches. Through careful examination of the coronary ostia and proximal segments, echocardiography can identify stenotic lesions, assess the degree of luminal narrowing, and detect potential complications such as plaque burden or thrombus formation. Additionally, echocardiography provides valuable information on left ventricular function and wall motion abnormalities, aiding in the overall assessment of cardiac performance. While coronary angiography remains the gold standard for definitive diagnosis, echocardiography serves as a non-invasive, radiation-free modality that complements the clinical evaluation, contributing to the comprehensive assessment of LMCAD and informing therapeutic decisions. Some studies have demonstrated good sensitivity and specificity for detection of LMCAD by using echocardiography. For instance, a study by Anjaneyulu *et al*. [16] evaluated the accuracy of TTE for evaluating LMCAD in 801 patients. The left main coronary artery and the adjacent segments of the LAD and the left circumflex (LCx) coronary artery were evaluated by color flow Doppler and corroborated with angiography. TTE demonstrated good sensitivity of 85% and positive predictive value of 82.5% for LMCAD detection in patients with abnormal doppler flow patterns. Additionally, TTE had high specificity, with 49 of 56 patients with a normal TTE doppler flow pattern having a normal left main on angiography (88% specificity, negative predictive value 90.7%).

TEE is also useful for evaluating for LMCAD. However, the studies showing its utility are from the 1990s and newer data is warranted. Nevertheless, these studies are worth mentioning. Alam *et al*. [17] conducted a study of 30 patients, of whom 10 had angiographically normal left main coronaries and 20 had stenotic left main coronary arteries. The TEE evaluation revealed no stenosis in 9 of 10 patients with normal left main trunks and detected atherosclerotic lesions in all 20 patients with stenotic left main trunks. Additional studies by Yoshida *et al*. [18] found TEE to be very sensitive (91%) and specific (100%) for the detection of proximal coronary stenoses. Similarly, the studies by Tardif *et al*. [19] and Khandheria *et al*. [20] found negative predictive accuracy of 98% and positive predictive accuracy of 100%. Moreover, the sensitivity and specificity were 100% for diagnosing proximal coronary stenosis when confirmed by angiography. However, it is worth noting that many of these studies are quite outdated and newer data in this area is needed.

### 3.3 Coronary Computed Tomography Angiography

Several other noninvasive techniques may be useful for the diagnosis of LMCAD. Such techniques are commonly utilized in the evaluation of chest pain and include exercise tolerance testing, stress echocardiography, nuclear medicine stress testing, myocardial perfusion imaging and CCTA. Different stress testing modalities are used to identify ischemia in the assessment of intermediate-risk chest pain and are recommended to be used in patients over the age of 65 or in the setting of high suspicion of obstructive CAD [5]. CCTA is often used in the evaluation of younger patients with a lower suspicion for obstructive disease, and is the only commonly used screening tool that is able to directly assess coronary anatomy and identify the location of obstructive disease [5]. Therefore, CCTA is the only non-invasive imaging modality that can directly diagnose significant LMCAD, which is defined as ≥50% stenosis of the left main coronary artery on both CCTA and invasive angiography [5, 21, 22].

CCTA is an effective tool for assessment of LMCAD when compared to invasive angiography. A meta-analysis by Paech *et al*. [23] demonstrated that CCTA has a 95.7% (95% CI 85.2–99.5) sensitivity and 97.1% (95% CI 95.7–98.1) specificity for diagnosing LMCAD when compared to conventional coronary angiography. The study also noted a negative predictive value of 100%, making CCTA quite an effective screening tool [23]. In an analysis of 1728 patients enrolled in the Initial Invasive or Conservative Strategy for Stable Coronary Disease (ISCHEMIA) trial, CCTA correctly identified the absence of significant left main stenosis in 97.1% of patients who later underwent invasive angiography [24]. However, this study was limited by the fact that CCTA analysis of LMCAD was only in those patients thought to be eligible for randomization after excluding LMCAD and in those randomized to the invasive strategy of therapy. There were 434 patients ineligible for randomization and, therefore, they did not have coronary angiography corroboration with CCTA. Additionally, the generalizability of this study comes into question because the population studied had a high pretest probability of having CAD. Nevertheless, CCTA has been found to perform well against intravascular ultrasound (IVUS), with a meta-analysis by Voros *et al*. [25] showing a sensitivity of 0.90 (95% CI, 0.83–0.94) and a specificity of 0.92 (95% CI, 0.90–0.93) for CCTA identification of coronary plaques in LMCAD when compared to IVUS. Additionally, CCTA been shown to generate similar heart team decisions when compared to invasive angiography in both left main and triple vessel disease and has a growing role in early identification and overall management [26]. CT-derived fractional flow reserve (FFR) is an additional non-invasive tool with CCTA, and has been shown to correlate with invasively measured FFR during angiography [27]. CT-derived FFR >0.80 is associated with positive short-term outcomes in patients with known LMCAD, and may become an effective tool to avoid additional invasive testing in the future [28].

CCTA continues to have roles outside of the identification of overall CAD and LMCAD. A study by Mieghem *et al*. [29] showed that CCTA could effectively rule out in-stent restenosis in patients with a prior PCI. CCTA may also be beneficial to stratify cardiovascular risk through other measurements and anatomic variants that could predispose to the formation of atherosclerotic plaques [30, 31].

### 3.4 Invasive Angiography and Intravascular Imaging

Invasive angiography remains the gold standard for diagnosis of LMCAD. Similar to the significant LMCAD definition for CCTA, significant left main stenosis on angiography is defined as ≥50% involvement of the diameter of the artery [21, 22]. In the setting of indeterminate left main lesions by angiography, additional intravascular techniques have been developed to determine lesion significance.

Both IVUS and optical coherence tomography (OCT) may be utilized in the diagnosis of indeterminate coronary lesions. IVUS is preferred to OCT in the evaluation of ostial left main lesions due to the fact that OCT requires clearance of blood, and the use of IVUS in the evaluation of indeterminate coronary lesions is a class 2 recommendation in both the American College of Cardiology (ACC) and European Society of Cardiology (ESC) guidelines [21, 32]. IVUS is used to assess intraluminal area, with studies showing that deferral of intervention is appropriate with a minimum diameter of ≥6 to 7.5 ${\mathrm{mm}}^{2}$ [33, 34]. The LITRO trial was a multicenter prospective study in Spain which showed that deferral of intervention of left main lesions with a minimum intraluminal diameter ≥6 ${\mathrm{mm}}^{2}$ was not associated with any increase in cardiac death, MI or revascularization [34]. De la Torre Hernandez *et al. * [34] also noted that only 4.4% of deferred patients went on to have left main intervention within the 2-year follow-up period. A meta-analysis by Cerrato *et al. * [35] compared outcomes based on deferral of intervention between IVUS and angiographic FFR in 908 cases of left main artery stenosis, showing similar incidence of major adverse cardiovascular events (MACEs) with rates of 5.1% and 6.4% in the FFR and IVUS groups, respectively.

FFR, the ratio of maximal blood flow distal to a lesion and maximal blood flow of the artery, is an additional diagnostic tool in the setting of invasive angiography [32]. FFR is the gold standard to determine functional significance of coronary stenosis, and current guidelines recommend the use of FFR to guide decision for intervention in angiographically ambiguous stenoses [21, 32]. A cutoff of ≤0.80 is considered significant and requires intervention per the ACC and ESC guidelines [21, 32]. An FFR cutoff of 0.80 is also used in recent studies evaluating FFR-guided intervention and is associated with decreased rates of major cardiovascular events when used to direct revascularization [36].

FFR can be used for the assessment and subsequent decision to revascularize or defer intervention of left main lesions. A meta-analysis of 6 cohort studies by Mallidi *et al*. [37] showed that the decision to revascularize based on FFR in intermediate left main lesions did not lead to any difference in major cardiovascular events, including MI, death and subsequent revascularization. An additional meta-analysis demonstrated FFR use to be associated with overall fewer stents and no change in adverse coronary events when compared to traditional angiography [38]. While this study evaluated lesions in all coronary arteries, there was no effect modification from patients included with left main disease. LMCAD was an exclusion criterion for several other notable trials showing benefit of FFR over conventional angiography, including the Proper Fractional Flow Reserve Criteria for Intermediate Lesions in the Era of Drug-Eluting Stent (DEFER-DES) and Fractional Flow Reserve versus Angiography for Multivessel Evaluation (FAME) trials [39, 40]. However, there are several limitations to FFR. FFR is generally effective in assessing the functional significance of coronary artery stenosis by measuring pressure differences across the lesion during hyperemia. However, when applied to LMCAD, its reliability may be compromised. The left main coronary artery is a major vessel supplying a substantial portion of the heart, and the involvement of such a critical artery poses challenges for FFR interpretation. The anatomical and hemodynamic complexity of LMCAD may lead to difficulties in achieving adequate hyperemia, impacting the accuracy of FFR measurements. Additionally, the long-term prognosis and appropriate treatment strategies for LMCAD may not be solely determined by FFR values, necessitating a comprehensive evaluation that combines clinical judgment, imaging modalities, and other physiological assessments to make informed decisions about patient management. The biggest concern for FFR assessment of LMCAD is the presence of distal coronary lesions to the proximal lesion, which can artificially increase the apparent FFR due to hemostasis distal to the lesion [41]. This is particularly challenging for left main lesions, which can have distal lesions in both the LAD and LCx. The left main coronary artery and subsequent downstream lesions function as serial lesions, potentially causing a reduction in the true flow across the left main coronary due to a severe stenosis downstream. This situation can lead to a false elevation of the left main FFR when measured in the unobstructed vessel [42]. Ultimately, achieving maximal hyperemia across the left main stenosis is crucial for an accurate FFR measurement. The total flow through the left main is determined by the cumulative flow in all daughter branches, with the magnitude of flow being proportional to the size of each artery’s viable myocardial bed.

## 4. LMCAD Pathophysiology and Genetic/Phenotypic Considerations

The left main coronary artery originates from the left aortic sinus and gives rise to the LAD and LCX arteries. In roughly one-third of patients, it also trifurcates with an intermediate ramus. Its average length is 10 mm (ranging from 2 to 23 mm), with a mean diameter of 3.9 ± 0.4 mm in women and 4.5 ± 0.5 mm in men. Structurally, it consists of three parts: the ostium, shaft, and distal segment. Of note, the left main ostium lacks an adventitial layer but contains abundant smooth muscle and elastic tissue, which may contribute to a unique response such as increased elastic recoil during PCI. Conversely, the left main shaft and distal segments have a tri-layered architecture like other epicardial vessels, with intima, media, and adventitia layers [43, 44].

Stenosis in the left main artery may occur ostially (23%), mid-shaft (15%) or distally (61%). The treatment strategies may differ depending on the location and severity of the disease. It is tantamount to recognize that LMCAD commonly coexists with multivessel CAD, as isolated LMCAD is only seen in about 4% to 6% of patients [44].

Atherosclerosis is a chronic, inflammatory, and fibroproliferative condition that primarily affects large and medium-sized arteries. Although the entire vascular system is exposed to systemic risk factors that contribute to atherogenesis (such as high cholesterol, smoking, high blood pressure, diabetes, chronic infections, and genetic predisposition), atherosclerotic lesions tend to form in specific areas of the arterial tree. These areas include near branch points, the outer wall of bifurcations, and the inner wall of curved regions, where there is irregular blood flow [45]. Local factors, including hemodynamic forces, play a crucial role in determining the location of atherosclerosis. These local hemodynamic forces encompass endothelial shear stress generated by blood flow and tensile stress from blood pressure. Of these forces, endothelial shear stress emerges as the most important factor in atherosclerosis development [46]. In the left main coronary artery, blood flow reaches its highest point during diastole, with a velocity of approximately 40–60 cm/s and a flow rate of about 200 mL/min/100 g. At the bifurcation of the left main coronary artery, shear forces reach their peak and create regions of high endothelial shear stress. Such physiology is pertinent to atherosclerosis in the left main coronary artery as the pathology of disease has been associated with the hemodynamics of blood flow. Atherosclerotic plaques tend to form in areas of low endothelial shear stress on the lateral wall of the bifurcation, opposite to the carina [46]. In contrast, the carina is often free from disease, likely due to the protective effect of high shear stress, which helps prevent plaque formation [43, 47]. The location and shape of stenosis are also affected by the size of the left main coronary artery. In shorter coronary arteries (<10 mm), stenotic areas are more commonly found near the origin rather than at the branching point (55% versus 38%). Conversely, in longer arteries, stenotic areas are predominantly found near the branching point rather than at the origin (18% at the origin versus 77% at the branching point) [48]. Additionally, stenotic areas near the origin tend to have negative remodeling, larger inner spaces, and less calcium compared to stenotic areas farther from the origin [43, 48].

Recent studies have shown that LMCAD, as determined by angiography, has a high level of heritability [49, 50]. Additionally, research has confirmed that asymptomatic siblings of patients with LMCAD are at a greater risk of future cardiovascular events compared to healthy siblings with other CAD phenotypes [51]. Various studies have also identified specific gene variants associated with LMCAD lesions [52, 53]. One such suggested gene variant is that of Cyclooxygenase-2 (COX-2). COX-2 is an isoform of cyclooxygenase that plays a role in the synthesis of eicosanoids, molecules involved in the pathogenesis of atherosclerosis [54, 55]. COX-2 expression is elevated in atherosclerotic lesions, particularly in macrophage and foam cells, indicating its significant involvement in atherosclerosis [56]. Previous animal studies have also shown that COX-2 promotes the formation of early atherosclerosis [57].

Based on the significance of COX-2, the prevailing hypothesis is that genetic variations in the *COX-2* gene factor into the heritability of LMCAD. To test this hypothesis, a study by Liu *et al*. [50] evaluated a hospital-based case-only cohort to detect and analyze three specific genetic variations (*COX-2* rs5277, rs5275, and rs689466) and their correlation with LMCAD. The results of this study provided evidence that a specific genetic variant, *COX-2* rs5277 C allele, was associated with an increased susceptibility to left main coronary artery lesions. Additionally, this variant correlated with a poorer prognosis among LMCAD patients who undergo CABG therapy [50]. However, it is worth noting this study was limited by selection bias due to being a single hospital study. Additionally, the correlation between COX-2 and LMCAD was based on just three SNPs, which limits the insights into the potential pathophysiological mechanisms.

All in all, the heritability of LMCAD remains an intriguing and largely unknown aspect within the realm of cardiovascular genetics. While it is established that genetics play a role in the development of CAD, the specific genetic factors contributing to the susceptibility and progression of LMCAD remain elusive. Limited studies have explored the heritability of LMCAD, and the intricate interplay between genetic predisposition and environmental factors is not yet fully understood. Further research is warranted to elucidate the specific genes and pathways associated with LMCAD.

### 4.1 LMCAD and Heart Failure

Patients with LMCAD face distinct considerations, particularly when they present with left ventricular (LV) systolic dysfunction and congestive heart failure (CHF). These conditions pose increased short-term risks for both percutaneous and surgical revascularization strategies. Observational studies have suggested that CABG may be more beneficial than PCI for patients with severe LV systolic dysfunction and LMCAD [44]. Nevertheless, these studies are limited by their observational nature, which predisposes them to significant biases. Additionally, there is data to support conservative management in some of these patients. For example, some studies had demonstrated that patients with left main stenoses of 50% to 70% or preserved left ventricular function had better survival rates with medical management alone (66% 3-year survival) compared to individuals with more severe LM disease (>70% stenosis) or reduced systolic function (41% 3-year survival) [58]. Of course, higher severity LMCAD carries a higher risk of complications and mortality, as evidenced in the literature. One study found that in patients with non-revascularized left main stenosis ≥70%, the 1-year survival rate ranged between 50% and 62% in the presence of resting chest pain, CHF, ST-T wave changes at rest, or left ventricular end-diastolic pressure >15 mm Hg. In contrast, patients without these clinical variables notably had a higher survival rate, ranging from 81% to 95%. Given the evidence in favor of CABG over PCI in these patients, contemporary practice patterns are skewed towards CABG. For instance, Maziak *et al.* [59] found that patients with left main stenosis of 75% or higher in individuals with New York Heart Association functional class IV heart failure had a higher likelihood of undergoing early CABG (within 10 days after coronary angiography). However, the advancements in mechanical circulatory support have shifted some attention towards PCI in patients with LMCAD and advanced CHF. The purpose of such support is to improve and maintain cardiac output to preserve systemic perfusion while unloading the left ventricle. These devices can thereby allow for PCIs with greater safety and precision. They may also reduce the risk of hemodynamic compromise. These technologies offer a vital bridge to recovery for patients with LMCAD and may potentially improve the success rate of PCI. However, randomized controlled trial (RCT) data is warranted in this area to further elucidate the potential for PCI in the LMCAD patient population.

### 4.2 LMCAD in the Elderly

The management and phenotypic presentation of LMCAD in elderly patients (age >75 years) warrants special considerations. Elderly patients often have reduced physiological reserves, making them more susceptible to the hemodynamic consequences of left main disease, including MI and heart failure. Additionally, older adults may experience atypical or silent symptoms, which can delay diagnosis and intervention. The management of LMCAD in this demographic requires a careful balance between the benefits of revascularization through PCI vs. CABG and the risks associated with advanced age, frailty, and potential complications.

Decisions regarding revascularization should be individualized to each patient profile of their overall comorbidities, functional status and life expectancy. Elderly patients tend to experience poorer postoperative outcomes following cardiac interventions, with higher rates of complications and mortality. As a result, many of these patients opt for PCI to expedite their recovery and reduce morbidity, even though it comes with a higher risk of requiring additional interventions within the next 5 to 10 years [60]. A British analysis highlighted the increasing complexity of lesions treated in the 2000s, particularly among octogenarians - a patient population that is the fastest growing group undergoing PCI [61]. About 46% of octogenarians had calcified lesions, and they also exhibited higher prevalence rates of tortuous lesions, ostial lesions, multi-vessel disease, and left main stenosis compared to patients under 80 years old. There was also significant increase over time in the number of octogenarians undergoing PCI for LMCAD. Despite these complexities, studies have shown that the clinical outcomes of octogenarians that undergo drug-eluting stents (DES) implantation for unprotected LMCAD are acceptable in the long term, as there were no significant differences in stroke, death or MI when compared to CABG [62].

### 4.3 Spontaneous Coronary Artery Dissection of the Left Main Coronary Artery

Spontaneous coronary artery dissection (SCAD) involving the left main coronary artery is a very rare and critical condition characterized by the separation of the arterial wall layers, which can lead to restricted blood flow to a significant portion of the heart. While SCAD typically occurs in smaller coronary arteries, when it affects the left main coronary artery, it presents unique challenges and risks. SCAD of the left main is often associated with a higher risk of MACEs, including MI and sudden cardiac death. The exact cause of SCAD remains unknown, but it is believed to be related to structural weaknesses in the arterial walls. Some predisposing factors include female gender, hormonal changes (such as those occurring during pregnancy or postpartum), connective tissue disorders, and underlying cardiovascular conditions.

SCAD only involves the left main coronary artery in about 4% of all SCAD cases [63]. However, there are higher incidences of SCAD occurring in the left main during pregnancy. In fact, this is the leading cause of MI among pregnant or postpartum patients. However, it constitutes a relatively small proportion of overall SCAD cases. More specifically, most SCAD events in these patients occur later in pregnancy, typically either in the third trimester or early postpartum period. Although, some cases have been reported to occur as early as 5 weeks of gestation and even as late as several months to a year or more postpartum [63, 64].

Because data on the optimal treatment strategies for SCAD in the left main are limited, the decision to treat conservatively versus invasively is heavily debated. Conservative management, including close monitoring and medical therapy, may be preferred for patients that are hemodynamically stable. Conversely, in patients with significant hemodynamic compromise or persistent ischemia from SCAD in the left main, invasive treatment may be preferable. A meta-analysis by Bocchino *et al*. [65] identified studies between 1990 and 2020 focused on comparing the effectiveness and safety of invasive revascularization versus medical therapy in the treatment of SCAD. The assessed endpoints included all-cause death, cardiovascular death, MI, CHF, recurrence of SCAD, and rates of target vessel revascularization. Ultimately, the study found that opting for a conservative approach in SCAD patients resulted in comparable clinical outcomes and lower rates of target vessel revascularization when contrasted with an invasive strategy. While this meta-analysis did not analyze left main SCAD patients specifically, it did rightly emphasize the consideration of conservative strategies in SCAD patients. Conservative management offers the benefit of avoiding procedural risks and promoting a tailored, patient-centered approach. Meanwhile, the evidence supporting revascularization in left main SCAD is also very limited and comes primarily from case reports, small case series, and observational studies. All in all, CABG may be considered as a treatment option for left main SCAD after a failed attempt at PCI, in cases of PCI complications, or when ischemia is refractory to conservative therapies [63, 66, 67].

Given the rarity and unique challenges posed by SCAD of the left main, a multidisciplinary approach involving cardiologists, interventional cardiologists, and cardiac surgeons is crucial for optimal patient care. Long-term follow-up and risk assessment are essential because SCAD can recur, emphasizing the importance of ongoing management and surveillance for these patients.

## 5. Society Guidelines for Left Main Disease

### 5.1 American Heart Association (AHA), American College of Cardiology (ACC) and Society for Cardiovascular Angiography and Interventions (SCAI)

Both the 2023 AHA/ACC Guidelines for the Management of Patients with Chronic Coronary Disease and the 2021 ACC/AHA/SCAI Guidelines for Coronary Artery Revascularization contain specific recommendations in the management of LMCAD and are reviewed below.

Both guidelines cite 50% stenosis diagnosed by invasive coronary angiography as significant for LMCAD [9, 32]. In situations where left main artery stenosis is ambiguous, IVUS can be used to further characterize the lesion with class 2a recommendation [9, 32]. While the 2021 guidelines cite evidence in support of deferment of revascularization in left main coronary arteries with minimal luminal area (MLA) of ≥6 to 7.5 ${\mathrm{mm}}^{2}$, the SCAI has previously published an MLA cutoff of >6 ${\mathrm{mm}}^{2}$ [68]. The authors also note that a smaller cutoff of 4.5–4.8 ${\mathrm{mm}}^{2}$ may be better suited for Asian patients due to smaller baseline coronary vessel size. In patients with indeterminant lesions and an FFR >0.80, the guidelines give a class 3 recommendation for PCI [32]. Additionally, invasive angiography for risk stratification is not recommended in patients with non-invasive testing consistent with left main disease (Class 3 recommendation, A level of evidence) [9].

Once significant LMCAD is diagnosed, both guidelines recommend CABG plus medical therapy over medical therapy alone with a class I recommendation [9, 32]. The authors also highlight the significance of involving a multidisciplinary heart team for the management of LMCAD and complex anatomical CAD, with involvement of general and interventional cardiologists as well as a cardiothoracic surgeon. The guidelines recommend consideration of PCI for left main revascularization and provide several scenarios where this may be appropriate. A class 2b recommendation is given for PCI in patients with low-to-intermediate risk anatomy (SYNTAX score ≤33) if stenting is able to provide equivalent revascularization to CABG [9, 32]. CABG is still preferred with a class 1 recommendation for patient with high anatomic complexity or significant multivessel CAD in addition to LMCAD [9, 32]. Elements that increase complexity of coronary anatomy include the presence of left main disease, trifurcation and complex bifurcation lesions, ostial left main disease and severe calcification. PCI can also be considered in patients who are poor candidates for surgery with a class 2a recommendation [9]. Reasons for poor surgical candidacy include poor distal targets for revascularization, severe left ventricular dysfunction or the presence of severe lung disease. CABG is generally preferred over PCI in diabetic patients, but PCI can be considered in patients with a SYNTAX score ≤33 (class 2b recommendation) [9, 32]. If PCI is performed for left main disease, IVUS guidance is accurate to assess stent expansion and is recommended to reduce ischemic events (class of recommendation 2a, level of evidence B-R) [22, 32]. OCT should not be used for stent implantation for ostial LMCAD [32]. While the above scenarios refer to stable ischemic heart disease, left main disease can lead to difficulty in the management of acute coronary syndrome. In the setting of ST-elevation myocardial infarction (STEMI), left main disease can be an anatomic limitation preventing PCI. Therefore, emergent CABG can be utilized as a reperfusion strategy to improve outcomes (class 2a) [32].

### 5.2 European Society of Cardiology (ESC) Guidelines

Recommendations specifically discussing management of LMCAD are also included in the European guidelines, specifically within the 2018 ESC and European Association for Cardiothoracic Surgery (EACTS) Guidelines on myocardial revascularization and 2019 ESC Guidelines for the diagnosis and management of chronic coronary syndromes.

Similar to the AHA/ACC/SCAI guidelines, the European guidelines define significant LMCAD as 50% stenosis by invasive coronary angiography [21]. The ESC guidelines also cite the same cutoffs for significant disease by both IVUS and FFR. The use of IVUS for the evaluation of intermediate LMCAD stenosis has a class IIa/b recommendation per the ESC, with some discordance between the chronic coronary syndrome and myocardial revascularization guidelines [21, 69]. Furthermore, the use of FFR is recommended for assessment of intermediate grade lesions (class I), noting that there are some limitations in left main disease with severe distal lesions [21].

The ESC guidelines also contain clear recommendations for the management of significant LMCAD. The authors support a Heart Team approach for decision making in complex unprotected left main disease. Revascularization is strongly recommended in significant LMCAD (class I recommendation; LOE A) [21]. The ESC guidelines recommend the stratification of patients by SYNTAX score to predict outcomes prior to PCI (class I). For patients with a low SYNTAX score (0–22), both PCI and CABG are given a level I recommendation [21]. For intermediate SYNTAX scores (23–32), CABG is given a class I recommendation while PCI is designated a class IIa recommendation. Lastly, for patients with SYNTAX scores ≥33 and significant LMCAD, CABG continues to carry a class I recommendation while PCI is rated as class III and not recommended. It is also worth mentioning that the ESC Guidelines recommend prioritization of completeness for revascularization in LMCAD when deciding between PCI and CABG (Class IIa, LOE B). A recent review of the 2018 guidelines by Byrne *et al*. [70] suggests that PCI is likely a reasonable alternative in low surgical risk patients and will likely have a class 2a recommendation in the upcoming 2024 chronic coronary syndrome guidelines. The ESC also provides guidance on surveillance following left main intervention, recommending consideration of repeat angiography to assess stent function at 3–12 months following PCI for unprotected LMCAD (class IIb, LOE C) [21].

The ESC guidelines also include supplementary information regarding quality insurance in the management of LMCAD. The ESC Guidelines give a class IIb recommendation that operators who perform left main PCI should have a case volume of 25 left main interventions per year. Additionally, they recommend that high-risk cases, such as left main intervention, should be performed at a center with a highly trained interventional cardiologist and access to mechanical support, as well as intensive care unit (ICU) capability [21].

### 5.3 Comparison between AHA/ACC/SCAI and ESC Guidelines

Overall, the AHA/ACC/SCAI and ESC guidelines share similar recommendations regarding management of LMCAD. The consensus regarding management of significant LMCAD is usually in favor of revascularization strategies over medical management since long term mortality rates are high for patients that are managed medically [71]. Both sets of guidelines are similar in terms of criteria and methods for diagnosis of LMCAD, with similar recommendations regarding when to utilize diagnostic testing such as invasive angiography, IVUS and FFR. However, these guidelines do notably differ on opinions regarding revascularization strategies in LMCAD. First, the ESC guidelines clearly stratify the indication for PCI based on SYNTAX score, while the AHA/ACC/SCAI guidelines are more subjective and rely more on heart team assessment of coronary complexity. The ESC guidelines also confer a class I recommendation for PCI in significant LMCAD with a low SYNTAX score, which drastically differs from the AHA/ACC/SCAI guidelines where PCI is given a 2a recommendation for low-moderate coronary complexity. The guidelines are more concordant in reference to intermediate and high complexity lesions, where both guidelines give a class 2a and 3 recommendations for PCI, respectively. Of note, both sets of guidelines highlight a heart team approach to make decisions regarding CABG vs. PCI in complex LMCAD and to assess additional risk factors. Lastly, the ESC guidelines highlight some additional recommendations regarding PCI volume and operator experience that is not included in the AHA/ACC/SCAI guidelines.

## 6. Clinical Trials and Evidence for CABG or PCI

The ACC/AHA/SCAI and ESC guideline recommendations for revascularization in LMCAD are based on evidence from several large-scale observational studies and trials.

### 6.1 CABG vs. Medical Therapy

The evidence for CABG over medical therapy in management of LMCAD comes from various studies. For instance, the Veterans Administration Coronary Artery Bypass Surgery Cooperative Study randomized 686 patients with stable CAD to a either medical therapy or revascularization with CABG and followed patients for roughly 11.2 years [72]. Of the enrolled patients, about 15% had significant LMCAD. The study found that at 42 months follow-up, CABG demonstrated a significant survival benefit over medical therapy in the patients with LMCAD. Additionally, a meta-analysis of RCTs comparing CABG with medical therapy supported these findings, demonstrating significantly lower mortality in the CABG group vs. medical treatment group at 5 years (10.2 vs. 15.8%; *p = *0.0001) for the group of patients with LMCAD [73]. Later studies have also supported this mortality benefit [74, 75, 76, 77, 78]. However, it is worth noting that most of this evidence in support of revascularization for LMCAD with CABG comes from older RCTs. Currently, there is no new data to refute this evidence due to the fact that contemporary clinical trials have excluded patients with significant LMCAD [79, 80].

### 6.2 PCI vs. Medical Therapy

While data is limited, there is some plausible evidence to support a mortality advantage for PCI revascularization over medical therapy for LMCAD. For example, a few observational studies have noted a significant mortality benefit of PCI over medical therapy in LMCAD patients [1, 81]. A meta-analysis of 19 studies on LMCAD observed a similar survival advantage for PCI over medical therapy that was comparable to the survival advantage of CABG over medical therapy [75]. All in all, further data on PCI in LMCAD is warranted. Regardless, opting for percutaneous revascularization presents a sensible choice for enhancing survival in specific patients with CAD of low to medium anatomic complexity and LMCAD. This holds true especially when the condition is equally amenable to either surgical or percutaneous revascularization, as opposed to relying solely on medical therapy.

### 6.3 PCI vs. CABG 

Given the significant evidence in support of revascularization over medical therapy for patients with LMCAD, PCI and CABG remain the leading choices of treatment. Historically, the mortality rate for medically treated LMCAD has been high at 73% over a 15-year period, leading to the recommendation of CABG for all stable ischemic heart disease patients to prevent fatal acute MI. However, advancements in medical technology, procedural techniques, antithrombotic agents, and medical therapy have made PCI an effective and legitimate alternative to CABG for LMCAD. With the use of DES reducing the risk of in-stent restenosis, PCI has gained recognition as a less invasive approach with favorable clinical outcomes for patients with unprotected LMCAD [1, 44].

Several RCTs and meta-analyses have compared the major outcomes of PCI versus CABG in patients with LMCAD (Table 3, Ref. [82, 83, 84, 85, 86, 87, 88, 89, 90, 91, 92, 93]). For instance, the Synergy between PCI with Taxus and Cardiac Surgery (SYNTAX) trial compared PCI with first generation Paclitaxel-eluting stents to CABG in patients with de-novo three vessel CAD including the left main coronary artery [82]. The trial included 705 patients with left main stenoses with a diverse range of disease complexity and followed various cardiovascular and mortality outcomes. The study randomly assigned patients to undergo revascularization using either CABG or PCI with DES and assessing the complexity of the disease through the SYNTAX score. Noteworthy findings emerged from the results. Across the entire patient cohort, the trial revealed comparable mortality rates between CABG and PCI, even in the extended 10-year follow-up of the SYNTAX trial, indicating no significant disparity in all-cause death between the two interventions [94]. However, a distinctive survival advantage was evident with CABG in patients presenting with three-vessel disease, whereas no such benefit was observed in those with LMCAD. A subsequent analysis delved into the angiographic severity of CAD, evaluated both visually and quantitatively using the SYNTAX score, and revealed that CABG conferred a survival advantage over PCI [83]. Specifically, individuals with diffuse CAD, as indicated by a high SYNTAX score of ≥33, exhibited a lower all-cause mortality rate after CABG compared to PCI [83, 94, 95]. Conversely, for patients with SYNTAX scores <33, there was no discernible difference in mortality rates between PCI and CABG. While these results are noteworthy, it is important to acknowledge that the SYNTAX trial included patients with only first-generation DESs, which may underestimate the efficacy of modern-day PCI and the use of newer generation DESs. Moreover, CABG patients were significantly undertreated with optimal medical therapy, which may not reflect real-world clinical treatment and outcomes.

**Table 3.** **RCTs and meta analyses comparing PCI vs. CABG for LMCAD**.

Study (Authors)	Trial name/Meta-analysis	N	Year	Results
Serruys *et al*. [82]	SYNTAX	705	2009	Rates of major adverse cardiac or cerebrovascular events at 12 months
				PCI: 17.8%
				CABG: 12.4% (*p* = 0.002)
Mäkikallio *et al*. [84]	NOBLE	1201	2016	5-year estimates
				MACCE
				PCI: 29%
				CABG: 19% (*p* = 0.0066)
				All-cause mortality:
				PCI: 12%
				CABG: 9% (*p* = 0.77)
				Non-procedural MI
				PCI: 7%
				CABG: 2% (*p* = 0.0040)
				Any revascularization
				PCI: 16%
				CABG: 10% (*p* = 0.032)
				Stroke
				PCI: 5%
				CABG: 2% (*p* = 0.073)
Stone *et al*. [85]	EXCEL	1905	2016	Composite of death from any cause, stroke, or myocardial infarction at 3 years
				PCI: 15.4%
				CABG: 14.7% (*p* = 0.02 for noninferiority)
Park *et al*. [86]	PRECOMBAT	600	2011	MACCE at 2 years
				PCI: 12.2%
				CABG: 8.1% (*p* = 0.12)
Head *et al*. [83]	Meta	11,518	2018	5-year all-cause mortality in LMCAD
				PCI: 10.7%
				CABG 10.5% (*p* = 0.52)
Kuno *et al*. [87]	Meta	4394	2020	All-cause death (HR)
				PCI: 1.11 [0.91–1.35]
				CABG: 1.11 [0.91–1.35], (*p* = 0.30)
Gallo *et al*. [88]	Meta	4595	2022	5-year outcomes
				MI (OR)
				PCI vs. CABG: 1.43 [1.13–1.79] (0.003)
				Repeat Revascularization (OR)
				PCI vs. CABG: 1.89 [1.58–2.26] (*p* < 0.001)
				Mortality and stroke rate did not differ at 5-year follow-up.
Ahmad *et al*. [89]	Meta	4612	2020	All-cause mortality [relative risk (RR)]
				PCI vs. CABG: 1.03, [0.81–1.32; *p* = 0.779]
				cardiac death
				PCI vs. CABG: 1.03 [95% CI 0.79–1.34; *p* = 0.817]
De Filippo *et al*. [90]	Meta	6296	2023	MACE or death for ostial/shaft LMCAD lesions
				CABG vs. PCI
				MACE: hazard ratio [HR], 1.0 [0.79–1.27]
				death: HR, 1.10 [95% CI, 0.84–1.46]
D’ascenzo *et al*. [91]	Meta	4394	2021	5-year all cause death
				CABG vs. PCI (OR): 0.93 [0.71–1.21]
Gaudino *et al*. [92]	Meta	13,260	2020	Pooled incidence rate ratio
				Cardiac mortality
				PCI: 0.96 (*p* = 0.06)
				Noncardiac mortality
				PCI: 1.41 (*p* = 0.15)
Buszman *et al*. [93]	LE MANS	105	2016	10-year results
				All-cause mortality
				PCI vs. CABG: 21.6% vs. 30.2%; (*p* = 0.41)
				MACCE
				PCI vs. CABG: 51.1% vs. 64.4%; (*p* = 0.28)
				MI
				PCI vs. CABG: 8.7 vs. 10.4%; (*p* = 0.62)

PCI, percutaneous coronary intervention; CABG, coronary artery bypass grafting; MACCE, major adverse cardiac or cerebrovascular event; OR, odds ratio; CI, confidence interval; HR, hazard ratio; NOBLE, Nordic-Baltic-British Left Main Revascularization Study; EXCEL, Effectiveness of Left Main Revascularization; PRECOMBAT, Patients with Left Main Coronary Artery Disease; LE MANS, Left Main Coronary Artery Stenting; MI, myocardial infarction; LMCAD, left main coronary artery disease; RCT, randomized controlled trial; N, number of patients.

The Nordic-Baltic-British Left Main Revascularization Study (NOBLE) trial represented another consequential investigation comparing PCI to CABG for LMCAD [84]. In this RCT involving 1201 LMCAD patients with an average SYNTAX score of 23, the outcomes were analyzed over a median follow-up period of approximately 3 years. Notably, the PCI group exhibited significantly higher rates of non-procedural MI, stroke, and repeat revascularization compared to the CABG group (29% vs. 19%; *p* = 0.007). Furthermore, the 5-year estimates of MACEs were 29% for PCI and 19% for CABG, surpassing the threshold for non-inferiority. Although no statistically significant disparity emerged in terms of all-cause mortality between CABG and PCI, the PCI approach was associated with markedly elevated rates of non-procedural MI, any revascularization, and stroke [84, 96]. However, this trial had some notable limitations. Firstly, the enrollment period spanned an extended duration, and the included participants may not accurately represent the broader population of individuals with LMCAD. Secondly, the inclusion criteria excluded clinically unstable patients. Moreover, a minority of individuals received first-generation DES, and those with acute coronary syndrome were predominantly prescribed clopidogrel and aspirin in lieu of other antiplatelet agents. In addition, the application of intravascular imaging varied, lacking predefined criteria for determining the optimal treatment.

The Evaluation of XIENCE versus Coronary Artery Bypass Surgery for Effectiveness of Left Main Revascularization (EXCEL) trial, which assessed patients with low or intermediate anatomical complexity of LMCAD, randomly assigned individuals to undergo revascularization with either PCI or CABG [85]. The findings, based on a median 3-year follow-up, revealed that PCI using everolimus-eluting stents was noninferior to CABG concerning the primary composite outcome of death, stroke, or MI. Although the PCI group experienced fewer periprocedural adverse events within 30 days, the CABG group had fewer postprocedural adverse events between 30 days and 3 years. Several limitations of the trial warrant consideration. Firstly, it was not feasible to blind patients and investigators to treatment assignments, potentially introducing event ascertainment bias. Secondly, despite the investigators specifically enrolling patients with low and intermediate SYNTAX scores, 24% of those randomized were found to have a high SYNTAX score based on angiographic core laboratory analysis. Thirdly, there was variation in long-term medication use after PCI and CABG, reflecting differences in practice between the two revascularization strategies. Lastly, this trial had relatively short follow-up and an extended follow-up is essential to assess whether further distinctions between PCI and CABG emerge over time.

In the Premier of Randomized Comparison of Bypass Surgery versus Angioplasty Using Sirolimus-Eluting Stent in Patients with Left Main Coronary Artery Disease (PRECOMBAT) trial, patients with unprotected left main coronary disease were randomly assigned to PCI with sirolimus-eluting stents or CABG [86]. The primary composite endpoint, major adverse cardiac or cerebrovascular events (MACCEs) [death, myocardial infarction, stroke, or ischemia-driven target-vessel revascularization], was assessed at 1 and 2-year follow-ups. PCI with sirolimus-eluting stents demonstrated noninferiority to CABG in terms of MACCEs and extended 10-year follow-up results did not show significant differences [97]. However, it is important to note that this study was underpowered due to the limited number of patients and low event rates.

While these trials provide valuable insights into the choice between CABG and PCI for LMCAD, it’s crucial to acknowledge limitations in evaluating patients with complex LMCAD. Trials and subsequent meta-analyses involving patients with low-to-medium anatomic complexity or LMCAD suitable for both surgical and percutaneous revascularization reported similar survival rates with PCI and CABG [84, 87, 88, 89, 96, 97, 98]. Notably, only the SYNTAX trial included patients with complex CAD (SYNTAX >32). In a recent pooled analysis of RCTs with over 11,000 patients, CABG demonstrated a significant survival benefit over PCI in the overall cohort [83]. However, among patients with LMCAD, there were similar risks for all-cause mortality at 5 years between PCI and CABG, with no significant differences in mortality based on SYNTAX scores. Despite limited inclusion of patients with high SYNTAX scores, a trend towards better survival with CABG was observed in this subgroup.

There are also more recent meta-analyses comparing various RCTs and observational studies on PCI vs. CABG for LMCAD worth mentioning. For instance, De Filippo *et al*. [90] compared MACE and all-cause death outcomes across 3 RCTs and 6 adjusted observational studies and found that in individuals with distal disease of the left main coronary artery, CABG was linked to a reduced occurrence of MACEs and revascularization when compared to PCI. However, no disparities in outcomes were noted for individuals with ostial or shaft disease of the left main. Another meta-analysis by D’Ascenzo *et al*. [91] included various RCTs that compared PCI to CABG and had a follow-up duration of at least 5 years. The primary endpoint was all-cause mortality and secondary endpoints encompassed MACCEs, including its individual components, and cardiovascular death. Among patients with LMCAD tracked over a 5-year period, there was no noteworthy distinction in all-cause and cardiovascular mortality between those who underwent PCI vs. CABG. However, CABG was associated with a diminished risk of MI, revascularization, and MACCEs, particularly in older patients and individuals with a high SYNTAX score.

To summarize, the current evidence suggests that PCI is a suitable alternative to CABG in LMCAD with low-to-intermediate anatomical complexity. Among patients with LMCAD and low anatomical complexity, there is indication that outcomes related to major clinical endpoints are comparable between PCI and CABG. However, for patients with high anatomical complexity of CAD and concurrent LMCAD, the limited number of participants in RCTs due to exclusion criteria makes risk estimates imprecise. Consequently, PCI for these patients is currently not endorsed, as reflected by a class III recommendation in both guidelines. Despite the scarcity of randomized trial data for this subgroup, ongoing advancements in PCI technology and techniques may yield promising results for individuals with LMCAD and complex anatomy. Thus, future studies should aim to assess the feasibility of PCI as an option for these patients.

Regardless of the optimal revascularization strategy planning, there is immense importance of the Heart Team. The Heart Team discussion holds paramount significance in managing complex scenarios, particularly in the context of LMCAD. This collaborative approach involves cardiovascular surgeons, interventional cardiologists, imaging specialists, and other relevant healthcare professionals working together to formulate the most effective treatment strategy for patients with intricate cardiac conditions. In the case of LMCAD, comorbidities play a pivotal role in shaping the optimal treatment plan. The presence of comorbid conditions such as diabetes, hypertension, or renal dysfunction significantly influences the risk-benefit profile of various interventions. Integrating comorbidity considerations into the decision-making process ensures a holistic approach to patient care, where the chosen treatment strategy not only addresses the coronary anatomy but also aligns with the patient’s overall health status. This comprehensive and multidisciplinary approach exemplifies the Heart Team’s commitment to delivering patient-centered care, enhancing treatment outcomes, and minimizing potential risks associated with complex cardiovascular cases.

## 7. Future Directions

### 7.1 The Issue of All-Cause Mortality vs. Cardiac Mortality

As previously discussed, there have been numerous trials comparing clinical outcomes for PCI with DES vs. CABG in patients with recent MI and stable ischemic heart disease. The trials evaluated mortality as a composite end point but were underpowered to differentiate between cardiac and non-cardiac mortality. The recent results of the 5-year evaluation of the EXCEL trial increased controversy as it demonstrated that PCI was associated with a significantly higher risk of all-cause mortality. However, that difference was not seen when comparing cardiac mortality alone. An analysis of PCI vs. CABG for left main disease compared the data obtained from the SYNTAX, PRECOMBAT, NOBLE, and EXCEL trials. The analysis concluded that patients with low to intermediate coronary anatomic complexity presenting with acute coronary syndrome at the time of left main revascularization were associated with a higher rate of early death. However, the rates of all-cause mortality were similar in PCI vs. CABG in this high-risk subgroup. In LMCAD, the cardiac mortality benefit for CABG over PCI was reduced and the primary difference between the two treatment modalities was due to a higher rate of noncardiac deaths with those that had PCI [92].

In LMCAD, however, the benefit of CABG over PCI for cardiac mortality was reduced, and the difference between the 2 revascularization modalities was mostly based on a higher rate of noncardiac deaths with those that underwent PCI.

### 7.2 Long Term Follow-Up

While the trials comparing CABG to PCI for LMCAD had several years’ worth of follow-up for outcomes, there is still a large need for evidence from trials and studies with longer-term follow up beyond 5 to 10 years. This is since long-term outcomes and prognoses of percutaneous treatment of LMCAD remain inconsistent. A recent meta-analysis by Wang *et al*. [53] compared the outcomes between medical therapy vs. DES vs. CABG and provided some insight into long term outcomes from RCTs. The study revealed a notable increase in the overall risk of MACCEs associated with PCI compared to CABG, primarily driven by a higher rate of revascularization. However, no significant differences were observed in terms of all-cause mortality, cardiac mortality, and recurrent MI. Interestingly, early PCI (within 30 days) was associated with a reduced risk of major adverse cardiac and cerebrovascular events compared to CABG. In the mixed-treatment comparison models, both CABG and DESs demonstrated improved survival compared to medical therapy, with no significant difference between them. Consequently, in patients with unprotected LMCAD, PCI with DESs exhibited similar all-cause and cardiac mortalities compared to CABG. Moreover, CABG was found to increase early (within 30 days) major adverse cardiac and cerebrovascular event rates, primarily due to an elevated risk of stroke. However, over more extended periods, PCI led to increased major adverse cardiac and cerebrovascular event rates, again driven by a higher incidence of recurrent revascularization.

10-year follow-up data comparing PCI efficacy is currently available via the Left Main Coronary Artery Stenting (LE MANS), PRECOMBAT and SYNTAX Extended Survival (SYNTAXES) trials which all indicate comparable outcomes of both strategies [93, 94, 97]. However, it has been noted that the above stated studies were underpowered due to small patient populations or unexpectedly low event rates. Two large RCTs that focused on LMCAD treatment and were sufficiently powered for non-inferiority testing of pre-specified major adverse cardiac and cerebrovascular events were the EXCEL and NOBLE trials. However, outcomes of both these trials, while contributing valuable data, generated significant controversy regarding the cardiac vs. non-cardiac mortality end points.

The EXCEL trial, in its five-year follow-up, revealed that PCI was comparable to CABG in terms of primary composite endpoints. Despite this, a closer examination of individual endpoints during the same period revealed a higher incidence of death from any cause in the PCI group. In contrast, the NOBLE trial reported that PCI was inferior to CABG in the short and mid-term follow-ups, with no significant impact on all-cause mortality. A meta-analysis incorporating data from these trials aimed to consolidate long-term follow-up outcomes, ultimately concluding that PCI and CABG exhibited similar safety profiles regarding all-cause mortality, MI, and stroke. However, patients treated with percutaneous interventions necessitated repeat revascularization [99].

The need for longer-term follow-up studies comparing PCI and CABG for LMCAD is paramount in advancing the understanding of the optimal treatment strategies for this critical condition. While existing research provides valuable insights into the short-to-medium-term outcomes of both interventions, the complexities of LMCAD warrant a more extended observation period. This is essential because cardiovascular events, such as restenosis, graft failure, and adverse clinical outcomes, may manifest years after the initial treatment. Longer-term studies would enable better assessment of the durability of PCI and CABG, potentially revealing trends and differences in late complications, overall survival, and quality of life, thereby guiding clinicians in making informed treatment decisions and improving the long-term prognosis for patients with LMCAD.

### 7.3 Role of SYNTAX Score

The purpose of the SYNTAX score is to risk stratify the patients that would potentially benefit the most from PCI vs. CABG by considering the anatomical extent and complexity of the CAD. The SYNTAX score is classified into 3 different tertiles: Tertile 1 is designated for low complexity lesions and anatomy with the lowest SYNTAX scores while Tertile 2 and 3 are reserved for patients with intermediate and high SYNTAX scores. In the meta-analysis conducted by Bundhun *et al*. [100] it was found that adverse outcomes were significantly greater with high SYNTAX scores (Tertile 2 + Tertile 3) as compared to low SYNTAX score (Tertile 1). Uniform findings were observed across all subgroups, encompassing patients with STEMI, non-ST elevation MI, LMCAD, and multivessel CAD. Notably, in a distinct analysis of patients with STEMI, a diminished SYNTAX score remained significantly linked to reduced adverse outcomes [100].

In 2019, the SYNTAX score 2 underwent a redevelopment. This updated scoring system utilizes key angiographic and clinical variables available at the time of decision-making to offer patients a personalized estimation of the potential treatment benefits of CABG versus PCI. The assessment includes predictions of a patient’s 50-year risk of experiencing a major adverse cardiovascular event and their 5- and 10-year risks of all-cause death. The SYNTAX III REVOLUTION trial demonstrated that clinical decision-making between CABG and PCI, when based on CCTA, exhibited comparable results and a high level of agreement with treatment decisions derived from angiography [101].

More recently, there is a new SYNTAX score available. In the SYNTAX III REVOLUTION trial [102], a pivotal focus was placed on evaluating outcomes following treatment through the development of a new CCTA-related SYNTAX score. The trial involved an international, multicenter investigation that randomly assigned two heart teams to decide on a treatment approach between PCIs and CABG. This decision-making process utilized either CCTA or conventional angiography. This innovative scoring system aimed to provide a comprehensive and precise assessment of CAD, thereby aiding in the determination of treatment efficacy. The trial was conducted with meticulous attention to this novel metric, utilizing advanced imaging techniques to gather detailed information about coronary anatomy. By incorporating the CCTA-related SYNTAX score, the trial sought to enhance the accuracy of outcome predictions, allowing for a more nuanced understanding of the impact of interventions on patients’ cardiovascular health. This strategic approach underscored the trial’s commitment to advancing both diagnostic and therapeutic paradigms in the management of CAD, ultimately contributing valuable insights to the medical community.

### 7.4 Dual Antiplatelet Therapy for LMCAD

Dual antiplatelet therapy (DAPT), composed of aspirin and a P2Y12 inhibitor, is the established standard of care post-PCI for patients with chronic coronary syndrome and acute coronary syndrome. However, the ideal duration of DAPT following PCI for LMCAD remains uncertain. Numerous research studies indicate that specific patient groups may benefit from either shortened or prolonged DAPT durations. The challenge with determining optimal DAPT duration arises from the fact that potential ischemic benefits associated with intensifying or extending DAPT are often counterbalanced by a simultaneous increase in the risk of bleeding. Questions also persist regarding the ideal DAPT duration after PCI and stenting in the left main coronary artery, particularly given the potentially severe consequences of stent thrombosis in this anatomical location. The available evidence on the trade-off between harm and benefit associated with varying DAPT durations after left main PCI is limited and subject to controversy. Moreover, these studies typically constitute only a small proportion, if any, of previous investigations into antiplatelet therapy for complex lesions, including LMCAD. In a recent retrospective analysis of patients who underwent PCI for LMCAD, the study aimed to evaluate the comparative efficacy and safety of extended-term (>12-month) DAPT versus 12-month or shortened DAPT [103]. Intriguingly, the decision to discontinue or continue DAPT after 12 months was left to individualized decision-making by treating physicians, weighing the patient’s risks of ischemia versus bleeding and considering patient preference. The primary outcome included a composite of death, MI, stent thrombosis, or stroke at 3 years. The study ultimately found that an individualized patient-tailored approach to longer duration (>12 months) of DAPT with aspirin plus a P2Y12 inhibitor (mostly clopidogrel) improved both composite and individual efficacy outcomes by reducing ischemic risk, without a concomitant increase in clinically relevant bleeding. A separate study by Choi *et al. * [104] reported similar findings after LMCAD interventions, with the DAPT >12 months group showing a lower net adverse clinical events rate than the DAPT ≤12 months group. Additionally, patients who maintained DAPT >12 months had lower rates of cardiac deaths, MI, and stent thromboses than those with DAPT <12 months, without increased major bleeding. Another retrospective study by Cho *et al*. [105] similarly found that DAPT durations <6 months or 6–12 months had a significantly higher adjusted hazard ratio for MACEs compared to DAPT for 12 to 24 months. However, evidence also suggests no benefit to extending DAPT duration after PCI for LMCAD, as shown in a post-hoc analysis of the EXCEL trial [106]. Overall, there is a significant lack of randomized trial data regarding optimal DAPT duration after left main PCI, and therefore, the choice of DAPT duration should be individualized, considering factors such as bleeding risk and patient-specific characteristics.

## 8. Conclusions

In conclusion, the choice between PCI and CABG for the management of LMCAD is a complex decision that hinges on various factors, including patient characteristics, anatomical considerations, and clinical context. While CABG offers a robust and time-tested strategy, particularly for patients with extensive CAD or complex anatomical features, there has been significant development over the course of the last two decades in the technology, technique, and imaging that is associated with PCI. PCI offers a less invasive option with favorable outcomes in certain scenarios. Of course, the decision should always remain individualized, considering the patient’s comorbidities, preferences, and the expertise of the healthcare team. However, ongoing research and advancements in PCI continue to refine the treatment landscape for LMCAD, making it imperative for clinicians to stay updated with the latest evidence-based guidelines to provide the most optimal care for their patients. Ultimately, the goal is to ensure the best possible outcomes, minimizing the risk of adverse events while improving the patient’s quality of life.
